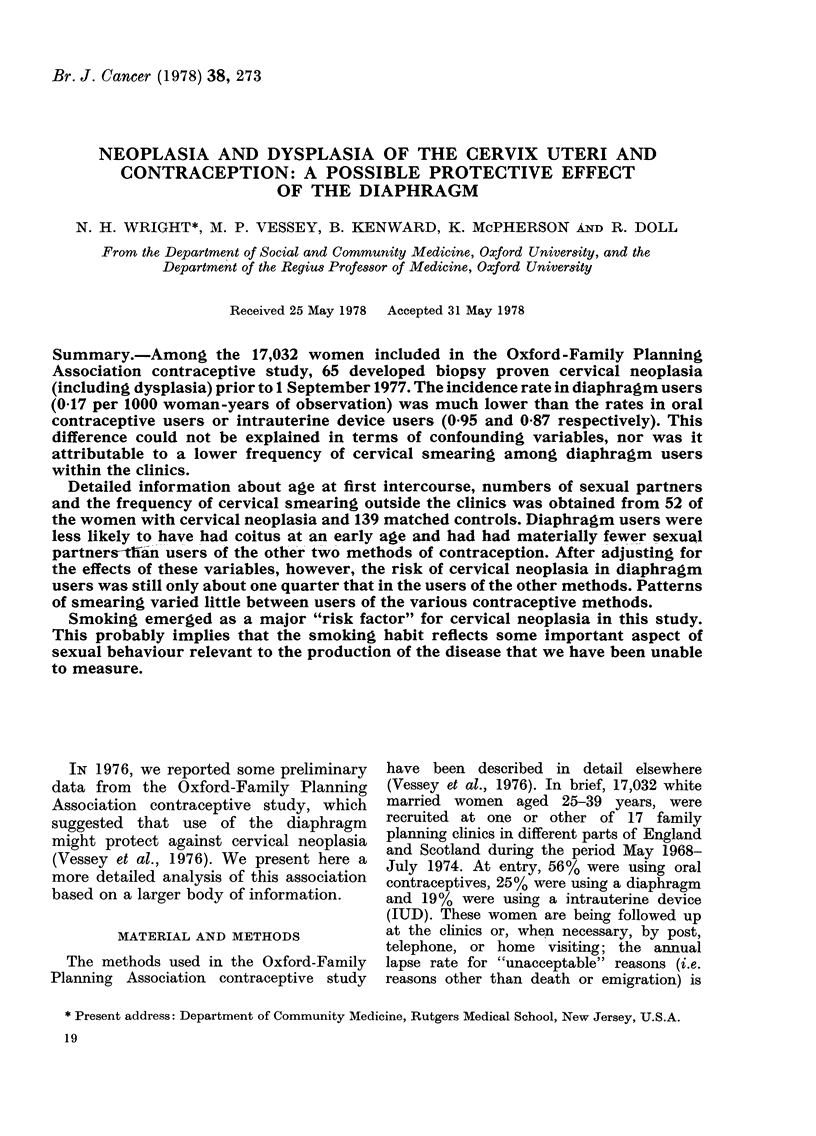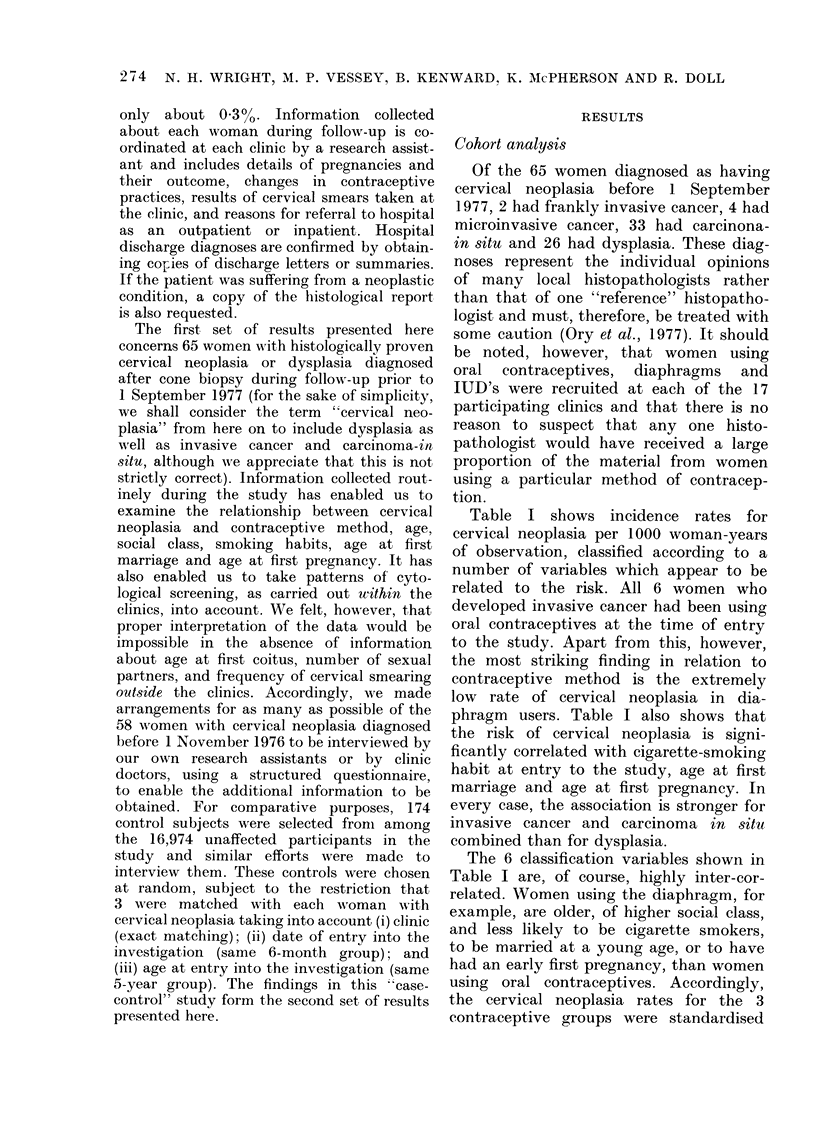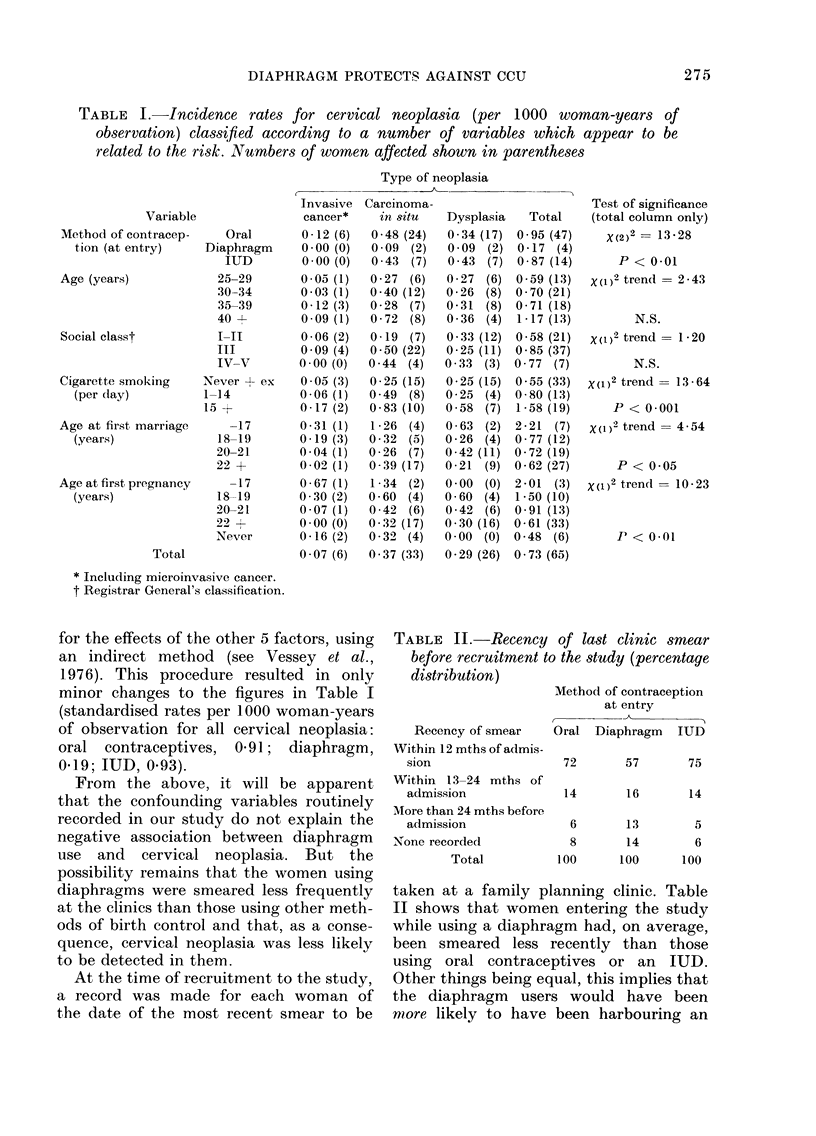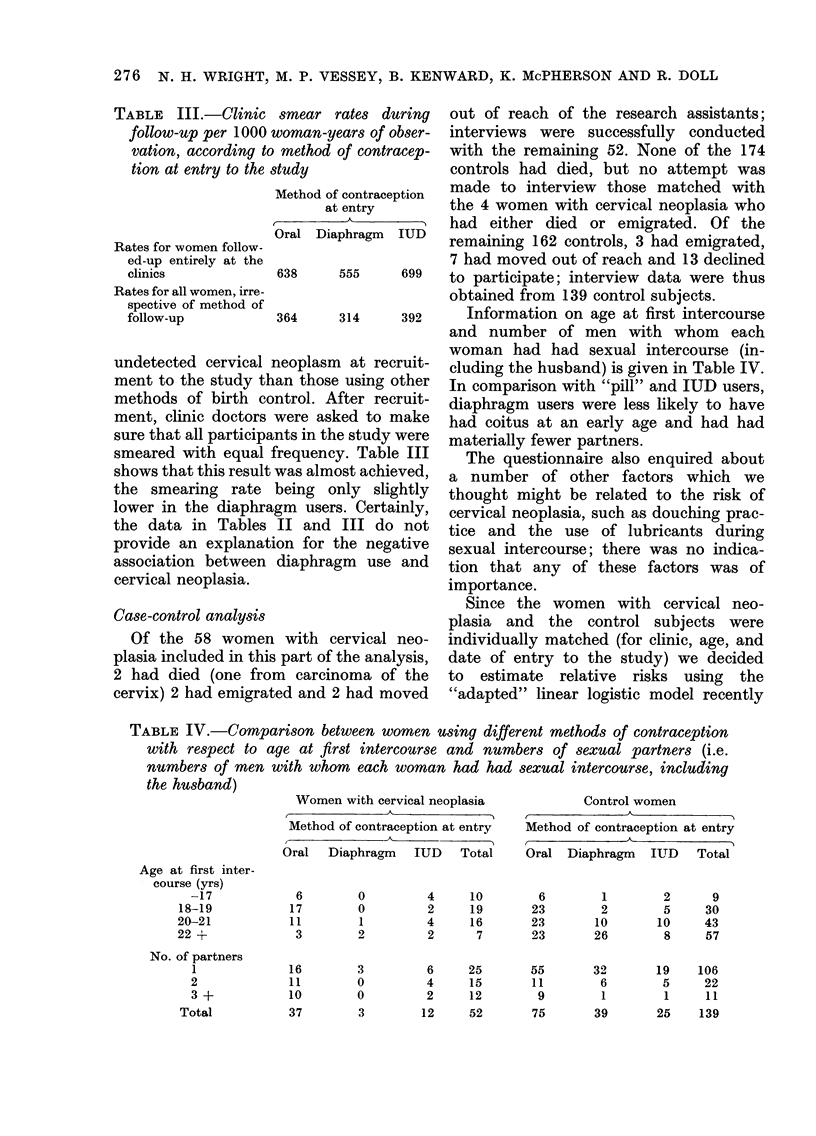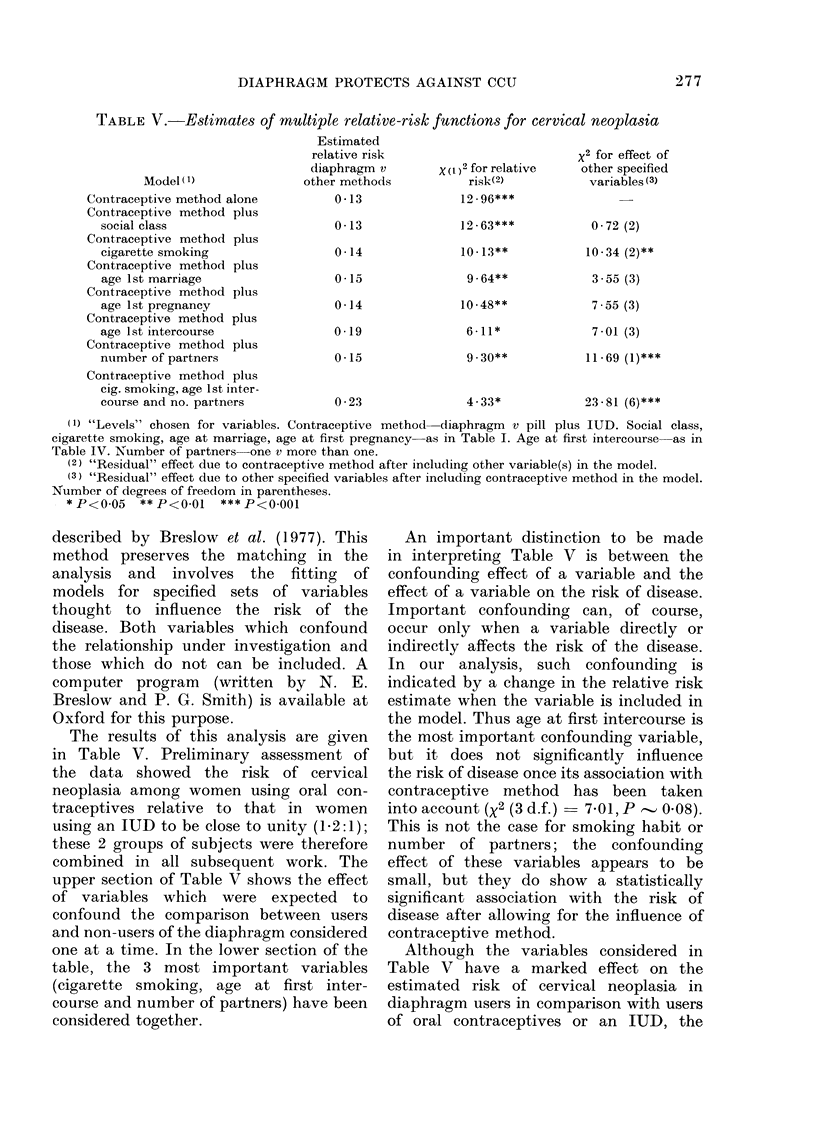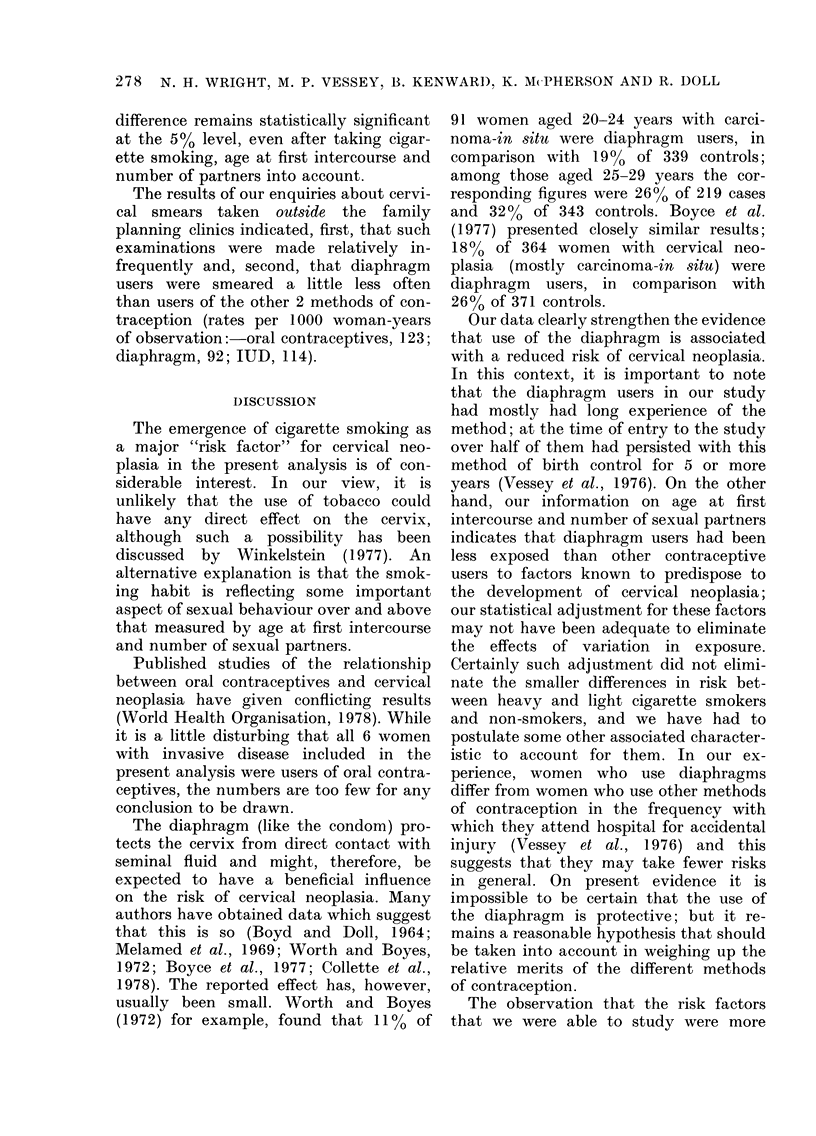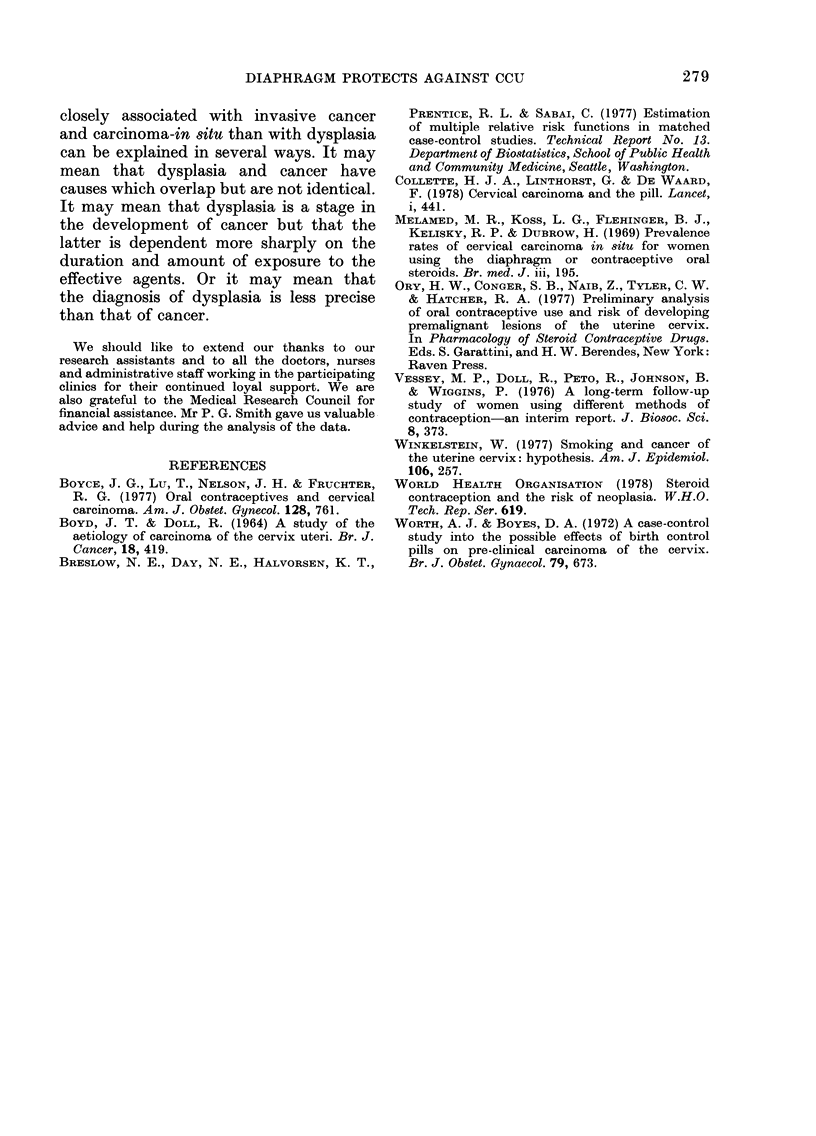# Neoplasia and dysplasia of the cervix uteri and contraception: a possible protective effect of the diaphragm.

**DOI:** 10.1038/bjc.1978.198

**Published:** 1978-08

**Authors:** N. H. Wright, M. P. Vessey, B. Kenward, K. McPherson, R. Doll

## Abstract

Among the 17,032 women included in the Oxford-Family Planning Association contraceptive study, 65 developed biopsy proven cervical neoplasia (including dysplasia) prior to 1 September 1977. The incidence rate in diaphragm users (0.17 per 1000 woman-years of observation) was much lower than the rates in oral contraceptive users or intrauterine device users (0.95 and 0.87 respectively). This difference could not be explained in terms of confounding variables, nor was it attributable to a lower frequency of cervical smearing among diaphragm users within the clinics. Detailed information about age at first intercourse, numbers of sexual partners and the frequency of cervical smearing outside the clinics was obtained from 52 of the women with cervical neoplasia and 139 matched controls. Diaphragm users were less likely to have had coitus at an early age and had had materially fewer sexual partners than users of the other two methods of contraception. After adjusting for the effects of these variables, however, the risk of cervical neoplasia in diaphragm users was still only about one quarter that in the users of the other methods. Patterns of smearing varied little between users of the various contraceptive methods. Smoking emerged as a major "risk factor" for cervical neoplasia in this study. This probably implies that the smoking habit reflects some important aspect of sexual behaviour relevant to the production of the disease that we have been unable to measure.


Br. J. Cancer (1978) 38, 273

### NEOPLASIA AND DYSPLASIA OF THE CERVIX UTERI AND CONTRACEPTION: A POSSIBLE PROTECTIVE EFFECT OF THE DIAPHRAGM

N. H. WRIGHT*, M. P. VESSEY, B. KENWARD, K. McPHERSON AND R. DOLL

*From the Department of Social and Community Medicine, Oxford University, and the Department of the Regius Professor of Medicine, Oxford University*

Received 25 May 1978 Accepted 31 May 1978

**Summary.—Among the 17,032 women included in the Oxford-Family Planning Association contraceptive study, 65 developed biopsy proven cervical neoplasia (including dysplasia) prior to 1 September 1977. The incidence rate in diaphragm users (0·17 per 1000 woman-years of observation) was much lower than the rates in oral contraceptive users or intrauterine device users (0·95 and 0·87 respectively). This difference could not be explained in terms of confounding variables, nor was it attributable to a lower frequency of cervical smearing among diaphragm users within the clinics.**

**Detailed information about age at first intercourse, numbers of sexual partners and the frequency of cervical smearing outside the clinics was obtained from 52 of the women with cervical neoplasia and 139 matched controls. Diaphragm users were less likely to have had coitus at an early age and had had materially fewer sexual partners than users of the other two methods of contraception. After adjusting for the effects of these variables, however, the risk of cervical neoplasia in diaphragm users was still only about one quarter that in the users of the other methods. Patterns of smearing varied little between users of the various contraceptive methods.**

**Smoking emerged as a major "risk factor" for cervical neoplasia in this study. This probably implies that the smoking habit reflects some important aspect of sexual behaviour relevant to the production of the disease that we have been unable to measure.**

In 1976, we reported some preliminary data from the Oxford-Family Planning Association contraceptive study, which suggested that use of the diaphragm might protect against cervical neoplasia (Vessey *et al.*, 1976). We present here a more detailed analysis of this association based on a larger body of information.

### MATERIAL AND METHODS

The methods used in the Oxford-Family Planning Association contraceptive study have been described in detail elsewhere (Vessey *et al.*, 1976). In brief, 17,032 white married women aged 25–39 years, were recruited at one or other of 17 family planning clinics in different parts of England and Scotland during the period May 1968–July 1974. At entry, 56% were using oral contraceptives, 25% were using a diaphragm and 19% were using a intrauterine device (IUD). These women are being followed up at the clinics or, when necessary, by post, telephone, or home visiting; the annual lapse rate for "unacceptable" reasons (*i.e.* reasons other than death or emigration) is

* Present address: Department of Community Medicine, Rutgers Medical School, New Jersey, U.S.A.

only about 0·3%. Information collected about each woman during follow-up is co-ordinated at each clinic by a research assistant and includes details of pregnancies and their outcome, changes in contraceptive practices, results of cervical smears taken at the clinic, and reasons for referral to hospital as an outpatient or inpatient. Hospital discharge diagnoses are confirmed by obtaining copies of discharge letters or summaries. If the patient was suffering from a neoplastic condition, a copy of the histological report is also requested.

The first set of results presented here concerns 65 women with histologically proven cervical neoplasia or dysplasia diagnosed after cone biopsy during follow-up prior to 1 September 1977 (for the sake of simplicity, we shall consider the term "cervical neoplasia" from here on to include dysplasia as well as invasive cancer and carcinoma-*in situ*, although we appreciate that this is not strictly correct). Information collected routinely during the study has enabled us to examine the relationship between cervical neoplasia and contraceptive method, age, social class, smoking habits, age at first marriage and age at first pregnancy. It has also enabled us to take patterns of cytological screening, as carried out *within* the clinics, into account. We felt, however, that proper interpretation of the data would be impossible in the absence of information about age at first coitus, number of sexual partners, and frequency of cervical smearing *outside* the clinics. Accordingly, we made arrangements for as many as possible of the 58 women with cervical neoplasia diagnosed before 1 November 1976 to be interviewed by our own research assistants or by clinic doctors, using a structured questionnaire, to enable the additional information to be obtained. For comparative purposes, 174 control subjects were selected from among the 16,974 unaffected participants in the study and similar efforts were made to interview them. These controls were chosen at random, subject to the restriction that 3 were matched with each woman with cervical neoplasia taking into account (i) clinic (exact matching); (ii) date of entry into the investigation (same 6-month group); and (iii) age at entry into the investigation (same 5-year group). The findings in this "case-control" study form the second set of results presented here.

### RESULTS

### *Cohort analysis*

Of the 65 women diagnosed as having cervical neoplasia before 1 September 1977, 2 had frankly invasive cancer, 4 had microinvasive cancer, 33 had carcinona-*in situ* and 26 had dysplasia. These diagnoses represent the individual opinions of many local histopathologists rather than that of one "reference" histopathologist and must, therefore, be treated with some caution (Ory *et al.*, 1977). It should be noted, however, that women using oral contraceptives, diaphragms and IUD's were recruited at each of the 17 participating clinics and that there is no reason to suspect that any one histopathologist would have received a large proportion of the material from women using a particular method of contraception.

Table I shows incidence rates for cervical neoplasia per 1000 woman-years of observation, classified according to a number of variables which appear to be related to the risk. All 6 women who developed invasive cancer had been using oral contraceptives at the time of entry to the study. Apart from this, however, the most striking finding in relation to contraceptive method is the extremely low rate of cervical neoplasia in diaphragm users. Table I also shows that the risk of cervical neoplasia is significantly correlated with cigarette-smoking habit at entry to the study, age at first marriage and age at first pregnancy. In every case, the association is stronger for invasive cancer and carcinoma *in situ* combined than for dysplasia.

The 6 classification variables shown in Table I are, of course, highly inter-correlated. Women using the diaphragm, for example, are older, of higher social class, and less likely to be cigarette smokers, to be married at a young age, or to have had an early first pregnancy, than women using oral contraceptives. Accordingly, the cervical neoplasia rates for the 3 contraceptive groups were standardised

TABLE I.—*Incidence rates for cervical neoplasia (per 1000 woman-years of observation) classified according to a number of variables which appear to be related to the risk. Numbers of women affected shown in parentheses*

| Variable | | Type of neoplasia: Invasive cancer* | Carcinoma-*in situ* | Dysplasia | Total | Test of significance (total column only) |
|---|---|---|---|---|---|---|
| Method of contraception (at entry) | Oral | 0·12 (6) | 0·48 (24) | 0·34 (17) | 0·95 (47) | $\chi_{(2)}^2 = 13·28$ |
| | Diaphragm | 0·00 (0) | 0·09 (2) | 0·09 (2) | 0·17 (4) | |
| | IUD | 0·00 (0) | 0·43 (7) | 0·43 (7) | 0·87 (14) | $P < 0·01$ |
| Age (years) | 25–29 | 0·05 (1) | 0·27 (6) | 0·27 (6) | 0·59 (13) | $\chi_{(1)}^2$ trend = 2·43 |
| | 30–34 | 0·03 (1) | 0·40 (12) | 0·26 (8) | 0·70 (21) | |
| | 35–39 | 0·12 (3) | 0·28 (7) | 0·31 (8) | 0·71 (18) | |
| | 40 + | 0·09 (1) | 0·72 (8) | 0·36 (4) | 1·17 (13) | N.S. |
| Social class† | I–II | 0·06 (2) | 0·19 (7) | 0·33 (12) | 0·58 (21) | $\chi_{(1)}^2$ trend = 1·20 |
| | III | 0·09 (4) | 0·50 (22) | 0·25 (11) | 0·85 (37) | |
| | IV–V | 0·00 (0) | 0·44 (4) | 0·33 (3) | 0·77 (7) | N.S. |
| Cigarette smoking (per day) | Never + ex | 0·05 (3) | 0·25 (15) | 0·25 (15) | 0·55 (33) | $\chi_{(1)}^2$ trend = 13·64 |
| | 1–14 | 0·06 (1) | 0·49 (8) | 0·25 (4) | 0·80 (13) | |
| | 15 + | 0·17 (2) | 0·83 (10) | 0·58 (7) | 1·58 (19) | $P < 0·001$ |
| Age at first marriage (years) | –17 | 0·31 (1) | 1·26 (4) | 0·63 (2) | 2·21 (7) | $\chi_{(1)}^2$ trend = 4·54 |
| | 18–19 | 0·19 (3) | 0·32 (5) | 0·26 (4) | 0·77 (12) | |
| | 20–21 | 0·04 (1) | 0·26 (7) | 0·42 (11) | 0·72 (19) | |
| | 22 + | 0·02 (1) | 0·39 (17) | 0·21 (9) | 0·62 (27) | $P < 0·05$ |
| Age at first pregnancy (years) | –17 | 0·67 (1) | 1·34 (2) | 0·00 (0) | 2·01 (3) | $\chi_{(1)}^2$ trend = 10·23 |
| | 18–19 | 0·30 (2) | 0·60 (4) | 0·60 (4) | 1·50 (10) | |
| | 20–21 | 0·07 (1) | 0·42 (6) | 0·42 (6) | 0·91 (13) | |
| | 22 + | 0·00 (0) | 0·32 (17) | 0·30 (16) | 0·61 (33) | |
| | Never | 0·16 (2) | 0·32 (4) | 0·00 (0) | 0·48 (6) | $P < 0·01$ |
| Total | | 0·07 (6) | 0·37 (33) | 0·29 (26) | 0·73 (65) | |

* Including microinvasive cancer.
† Registrar General's classification.

for the effects of the other 5 factors, using an indirect method (see Vessey *et al.*, 1976). This procedure resulted in only minor changes to the figures in Table I (standardised rates per 1000 woman-years of observation for all cervical neoplasia: oral contraceptives, 0·91; diaphragm, 0·19; IUD, 0·93).

From the above, it will be apparent that the confounding variables routinely recorded in our study do not explain the negative association between diaphragm use and cervical neoplasia. But the possibility remains that the women using diaphragms were smeared less frequently at the clinics than those using other methods of birth control and that, as a consequence, cervical neoplasia was less likely to be detected in them.

At the time of recruitment to the study, a record was made for each woman of the date of the most recent smear to be taken at a family planning clinic. Table II shows that women entering the study while using a diaphragm had, on average, been smeared less recently than those using oral contraceptives or an IUD. Other things being equal, this implies that the diaphragm users would have been *more* likely to have been harbouring an

TABLE II.—*Recency of last clinic smear before recruitment to the study (percentage distribution)*

| Recency of smear | Method of contraception at entry: Oral | Diaphragm | IUD |
|---|---|---|---|
| Within 12 mths of admission | 72 | 57 | 75 |
| Within 13–24 mths of admission | 14 | 16 | 14 |
| More than 24 mths before admission | 6 | 13 | 5 |
| None recorded | 8 | 14 | 6 |
| Total | 100 | 100 | 100 |

TABLE III.—*Clinic smear rates during follow-up per* 1000 *woman-years of observation, according to method of contraception at entry to the study*

| | Method of contraception at entry | | |
|---|---|---|---|
| | Oral | Diaphragm | IUD |
| Rates for women followed-up entirely at the clinics | 638 | 555 | 699 |
| Rates for all women, irrespective of method of follow-up | 364 | 314 | 392 |

undetected cervical neoplasm at recruitment to the study than those using other methods of birth control. After recruitment, clinic doctors were asked to make sure that all participants in the study were smeared with equal frequency. Table III shows that this result was almost achieved, the smearing rate being only slightly lower in the diaphragm users. Certainly, the data in Tables II and III do not provide an explanation for the negative association between diaphragm use and cervical neoplasia.

*Case-control analysis*

Of the 58 women with cervical neoplasia included in this part of the analysis, 2 had died (one from carcinoma of the cervix) 2 had emigrated and 2 had moved out of reach of the research assistants; interviews were successfully conducted with the remaining 52. None of the 174 controls had died, but no attempt was made to interview those matched with the 4 women with cervical neoplasia who had either died or emigrated. Of the remaining 162 controls, 3 had emigrated, 7 had moved out of reach and 13 declined to participate; interview data were thus obtained from 139 control subjects.

Information on age at first intercourse and number of men with whom each woman had had sexual intercourse (including the husband) is given in Table IV. In comparison with "pill" and IUD users, diaphragm users were less likely to have had coitus at an early age and had had materially fewer partners.

The questionnaire also enquired about a number of other factors which we thought might be related to the risk of cervical neoplasia, such as douching practice and the use of lubricants during sexual intercourse; there was no indication that any of these factors was of importance.

Since the women with cervical neoplasia and the control subjects were individually matched (for clinic, age, and date of entry to the study) we decided to estimate relative risks using the "adapted" linear logistic model recently

TABLE IV.—*Comparison between women using different methods of contraception with respect to age at first intercourse and numbers of sexual partners* (i.e. *numbers of men with whom each woman had had sexual intercourse, including the husband*)

| | Women with cervical neoplasia | | | | Control women | | | |
|---|---|---|---|---|---|---|---|---|
| | Method of contraception at entry | | | | Method of contraception at entry | | | |
| | Oral | Diaphragm | IUD | Total | Oral | Diaphragm | IUD | Total |
| Age at first intercourse (yrs) | | | | | | | | |
| –17 | 6 | 0 | 4 | 10 | 6 | 1 | 2 | 9 |
| 18–19 | 17 | 0 | 2 | 19 | 23 | 2 | 5 | 30 |
| 20–21 | 11 | 1 | 4 | 16 | 23 | 10 | 10 | 43 |
| 22 + | 3 | 2 | 2 | 7 | 23 | 26 | 8 | 57 |
| No. of partners | | | | | | | | |
| 1 | 16 | 3 | 6 | 25 | 55 | 32 | 19 | 106 |
| 2 | 11 | 0 | 4 | 15 | 11 | 6 | 5 | 22 |
| 3 + | 10 | 0 | 2 | 12 | 9 | 1 | 1 | 11 |
| Total | 37 | 3 | 12 | 52 | 75 | 39 | 25 | 139 |

TABLE V.—*Estimates of multiple relative-risk functions for cervical neoplasia*

| Model (1) | Estimated relative risk diaphragm *v* other methods | $\chi_{(1)}^2$ for relative risk (2) | $\chi^2$ for effect of other specified variables (3) |
|---|---|---|---|
| Contraceptive method alone | 0·13 | 12·96*** | — |
| Contraceptive method plus social class | 0·13 | 12·63*** | 0·72 (2) |
| Contraceptive method plus cigarette smoking | 0·14 | 10·13** | 10·34 (2)** |
| Contraceptive method plus age 1st marriage | 0·15 | 9·64** | 3·55 (3) |
| Contraceptive method plus age 1st pregnancy | 0·14 | 10·48** | 7·55 (3) |
| Contraceptive method plus age 1st intercourse | 0·19 | 6·11* | 7·01 (3) |
| Contraceptive method plus number of partners | 0·15 | 9·30** | 11·69 (1)*** |
| Contraceptive method plus cig. smoking, age 1st intercourse and no. partners | 0·23 | 4·33* | 23·81 (6)*** |

(1) "Levels" chosen for variables. Contraceptive method—diaphragm *v* pill plus IUD. Social class, cigarette smoking, age at marriage, age at first pregnancy—as in Table I. Age at first intercourse—as in Table IV. Number of partners—one *v* more than one.
(2) "Residual" effect due to contraceptive method after including other variable(s) in the model.
(3) "Residual" effect due to other specified variables after including contraceptive method in the model. Number of degrees of freedom in parentheses.
* $P<0{\cdot}05$ ** $P<0{\cdot}01$ *** $P<0{\cdot}001$

described by Breslow *et al.* (1977). This method preserves the matching in the analysis and involves the fitting of models for specified sets of variables thought to influence the risk of the disease. Both variables which confound the relationship under investigation and those which do not can be included. A computer program (written by N. E. Breslow and P. G. Smith) is available at Oxford for this purpose.

The results of this analysis are given in Table V. Preliminary assessment of the data showed the risk of cervical neoplasia among women using oral contraceptives relative to that in women using an IUD to be close to unity (1·2:1); these 2 groups of subjects were therefore combined in all subsequent work. The upper section of Table V shows the effect of variables which were expected to confound the comparison between users and non-users of the diaphragm considered one at a time. In the lower section of the table, the 3 most important variables (cigarette smoking, age at first intercourse and number of partners) have been considered together.

An important distinction to be made in interpreting Table V is between the confounding effect of a variable and the effect of a variable on the risk of disease. Important confounding can, of course, occur only when a variable directly or indirectly affects the risk of the disease. In our analysis, such confounding is indicated by a change in the relative risk estimate when the variable is included in the model. Thus age at first intercourse is the most important confounding variable, but it does not significantly influence the risk of disease once its association with contraceptive method has been taken into account ($\chi^2$ (3 d.f.) $= 7{\cdot}01, P \sim 0{\cdot}08$). This is not the case for smoking habit or number of partners; the confounding effect of these variables appears to be small, but they do show a statistically significant association with the risk of disease after allowing for the influence of contraceptive method.

Although the variables considered in Table V have a marked effect on the estimated risk of cervical neoplasia in diaphragm users in comparison with users of oral contraceptives or an IUD, the difference remains statistically significant at the 5% level, even after taking cigarette smoking, age at first intercourse and number of partners into account.

The results of our enquiries about cervical smears taken *outside* the family planning clinics indicated, first, that such examinations were made relatively infrequently and, second, that diaphragm users were smeared a little less often than users of the other 2 methods of contraception (rates per 1000 woman-years of observation:—oral contraceptives, 123; diaphragm, 92; IUD, 114).

### DISCUSSION

The emergence of cigarette smoking as a major "risk factor" for cervical neoplasia in the present analysis is of considerable interest. In our view, it is unlikely that the use of tobacco could have any direct effect on the cervix, although such a possibility has been discussed by Winkelstein (1977). An alternative explanation is that the smoking habit is reflecting some important aspect of sexual behaviour over and above that measured by age at first intercourse and number of sexual partners.

Published studies of the relationship between oral contraceptives and cervical neoplasia have given conflicting results (World Health Organisation, 1978). While it is a little disturbing that all 6 women with invasive disease included in the present analysis were users of oral contraceptives, the numbers are too few for any conclusion to be drawn.

The diaphragm (like the condom) protects the cervix from direct contact with seminal fluid and might, therefore, be expected to have a beneficial influence on the risk of cervical neoplasia. Many authors have obtained data which suggest that this is so (Boyd and Doll, 1964; Melamed *et al.*, 1969; Worth and Boyes, 1972; Boyce *et al.*, 1977; Collette *et al.*, 1978). The reported effect has, however, usually been small. Worth and Boyes (1972) for example, found that 11% of 91 women aged 20–24 years with carcinoma-*in situ* were diaphragm users, in comparison with 19% of 339 controls; among those aged 25–29 years the corresponding figures were 26% of 219 cases and 32% of 343 controls. Boyce *et al.* (1977) presented closely similar results; 18% of 364 women with cervical neoplasia (mostly carcinoma-*in situ*) were diaphragm users, in comparison with 26% of 371 controls.

Our data clearly strengthen the evidence that use of the diaphragm is associated with a reduced risk of cervical neoplasia. In this context, it is important to note that the diaphragm users in our study had mostly had long experience of the method; at the time of entry to the study over half of them had persisted with this method of birth control for 5 or more years (Vessey *et al.*, 1976). On the other hand, our information on age at first intercourse and number of sexual partners indicates that diaphragm users had been less exposed than other contraceptive users to factors known to predispose to the development of cervical neoplasia; our statistical adjustment for these factors may not have been adequate to eliminate the effects of variation in exposure. Certainly such adjustment did not eliminate the smaller differences in risk between heavy and light cigarette smokers and non-smokers, and we have had to postulate some other associated characteristic to account for them. In our experience, women who use diaphragms differ from women who use other methods of contraception in the frequency with which they attend hospital for accidental injury (Vessey *et al.*, 1976) and this suggests that they may take fewer risks in general. On present evidence it is impossible to be certain that the use of the diaphragm is protective; but it remains a reasonable hypothesis that should be taken into account in weighing up the relative merits of the different methods of contraception.

The observation that the risk factors that we were able to study were more closely associated with invasive cancer and carcinoma-*in situ* than with dysplasia can be explained in several ways. It may mean that dysplasia and cancer have causes which overlap but are not identical. It may mean that dysplasia is a stage in the development of cancer but that the latter is dependent more sharply on the duration and amount of exposure to the effective agents. Or it may mean that the diagnosis of dysplasia is less precise than that of cancer.

We should like to extend our thanks to our research assistants and to all the doctors, nurses and administrative staff working in the participating clinics for their continued loyal support. We are also grateful to the Medical Research Council for financial assistance. Mr P. G. Smith gave us valuable advice and help during the analysis of the data.

### REFERENCES

BOYCE, J. G., LU, T., NELSON, J. H. & FRUCHTER, R. G. (1977) Oral contraceptives and cervical carcinoma. *Am. J. Obstet. Gynecol.* **128**, 761.

BOYD, J. T. & DOLL, R. (1964) A study of the aetiology of carcinoma of the cervix uteri. *Br. J. Cancer*, **18**, 419.

BRESLOW, N. E., DAY, N. E., HALVORSEN, K. T., PRENTICE, R. L. & SABAI, C. (1977) Estimation of multiple relative risk functions in matched case-control studies. *Technical Report No. 13. Department of Biostatistics, School of Public Health and Community Medicine, Seattle, Washington.*

COLLETTE, H. J. A., LINTHORST, G. & DE WAARD, F. (1978) Cervical carcinoma and the pill. *Lancet*, i, 441.

MELAMED, M. R., KOSS, L. G., FLEHINGER, B. J., KELISKY, R. P. & DUBROW, H. (1969) Prevalence rates of cervical carcinoma *in situ* for women using the diaphragm or contraceptive oral steroids. *Br. med. J.* iii, 195.

ORY, H. W., CONGER, S. B., NAIB, Z., TYLER, C. W. & HATCHER, R. A. (1977) Preliminary analysis of oral contraceptive use and risk of developing premalignant lesions of the uterine cervix. In *Pharmacology of Steroid Contraceptive Drugs.* Eds. S. Garattini, and H. W. Berendes, New York: Raven Press.

VESSEY, M. P., DOLL, R., PETO, R., JOHNSON, B. & WIGGINS, P. (1976) A long-term follow-up study of women using different methods of contraception—an interim report. *J. Biosoc. Sci.* **8**, 373.

WINKELSTEIN, W. (1977) Smoking and cancer of the uterine cervix: hypothesis. *Am. J. Epidemiol.* **106**, 257.

WORLD HEALTH ORGANISATION (1978) Steroid contraception and the risk of neoplasia. *W.H.O. Tech. Rep. Ser.* **619**.

WORTH, A. J. & BOYES, D. A. (1972) A case-control study into the possible effects of birth control pills on pre-clinical carcinoma of the cervix. *Br. J. Obstet. Gynaecol.* **79**, 673.